# Laser induced mortality of *Anopheles stephensi* mosquitoes

**DOI:** 10.1038/srep20936

**Published:** 2016-02-18

**Authors:** Matthew D. Keller, David J. Leahy, Bryan J. Norton, Threeric Johanson, Emma R. Mullen, Maclen Marvit, Arty Makagon

**Affiliations:** 1 Intellectual Ventures Laboratory, Bellevue, Washington, 98007, United States of America

## Abstract

Small, flying insects continue to pose great risks to both human health and agricultural production throughout the world, so there remains a compelling need to develop new vector and pest control approaches. Here, we examined the use of short (<25 ms) laser pulses to kill or disable anesthetized female *Anopheles stephensi* mosquitoes, which were chosen as a representative species. The mortality of mosquitoes exposed to laser pulses of various wavelength, power, pulse duration, and spot size combinations was assessed 24 hours after exposure. For otherwise comparable conditions, green and far-infrared wavelengths were found to be more effective than near- and mid-infrared wavelengths. Pulses with larger laser spot sizes required lower lethal energy densities, or fluence, but more pulse energy than for smaller spot sizes with greater fluence. Pulse duration had to be reduced by several orders of magnitude to significantly lower the lethal pulse energy or fluence required. These results identified the most promising candidates for the lethal laser component in a system being designed to identify, track, and shoot down flying insects in the wild.

Worldwide, mosquitoes are responsible for nearly 1 million deaths each year by transmitting approximately 100 different diseases^1^. Chief among them is malaria, which infects about 200 million and kills 200,000 people annually, mostly children under age five^2^,^3^,^4^. Mosquitoes are also vectors of yellow fever, which is fatal in 30,000 to 80,000 of the roughly 200,000 annual cases^5^,^6^,^7^; of dengue fever, whose incidence is rapidly on the rise and infects 50 to 100 million people per year, with around 20,000 deaths^8^,^9^; and for numerous other diseases like chikungunya^10^,^11^, West Nile^12^,^13^, and various other encephalitic conditions^14^,^15^.

Besides the toll on humans, mosquitoes and other similarly sized insects are major agricultural pests. Livestock can be infected by the same or closely related pathogens as infect humans, leading to heavy losses^1^,^16^. Dairy production is negatively impacted by nuisance insects^17^,^18^, which may cost the US dairy industry upwards of $350 million per year^17^. Fruit infestations by pests such as the Queensland^19^,^20^ and Mediterranean^21^ fruit flies (Qfly and medfly, respectively), spotted wing *Drosophila*^22^, and Asian citrus psyllid^23^ can wreak havoc on a region’s produce industry. For example, the Qfly costs Australian growers up to $50 million each year in produce lost both to Qfly-induced damage and to restrictions on exports from Qfly-endemic regions^19^,^20^.

Recent large-scale vector control efforts have been met with mixed success. In particular, the work by African governments and international organizations to distribute insecticide-treated nets (ITNs) and to promote indoor residual spraying (IRS) have reduced malaria deaths to approximately 1/2 to 1/3 the peak number of 1.8 million in 2004^4^. The benefits of these efforts appear to have leveled off, however, as ITN’s only work indoors and often get damaged or repurposed, leading to modest overall usage in some endemic regions^2^. Further hindering these efforts is the trend for mosquitoes to become increasingly resistant to the insecticides used^24^,^25^. Although new insecticides are under development, their long-term safety and efficacy are unclear^26^. The use of chemical pesticides for commercial agricultural applications is a controversial topic among consumers as well.

There is thus a need for new, innovative methods in pest and vector control to complement existing core techniques. Optical approaches have been gaining popularity for identifying insects and studying their behavior^27^,^28^,^29^,^30^,^31^,^32^, providing information not readily available from conventional monitoring approaches like traps and human-landing catches. A recent report also demonstrated that exposure to low power blue light for several hours to days could disable insects such as fruit flies and mosquitoes^33^. At Intellectual Ventures Laboratory (IV Lab), the Photonic Fence (PF) has been proposed as a device that could both detect and disable flying insects using optical technology. At a high level, the system would work by first using a camera system with near-infrared illumination to identify target insects in an active region. It would discriminate targets from non-targets based on size and, if needed, wingbeat frequency^27^ and/or contextual information like time of day. Once a target is identified, the system would then direct a brief (no more than tens of milliseconds) pulse of laser light to kill or otherwise disable the insect. As currently envisioned, a single active region would be approximately 3 meters tall, less than a meter wide, and 30 to 100 meters long. By placing a single unit between known insect breeding grounds or nesting locations and the desired area of protection, or by using multiple units to create an enclosure, the PF could prevent or minimize crop damage or disease spread in local areas. It could do so without compromising the eye safety of people nearby through the use of a backstop to absorb any laser pulses not hitting a target, and with a separate object-detecting system that ensures no people or other large animals can be exposed to potentially dangerous lateral reflections; the size of this lateral “no-fire zone” varies according to the exact laser pulse parameters, which are discussed below.

We have preliminary evidence that the various sub-systems of the proposed PF can work as envisioned. The work presented here was focused on testing the lethal laser component in isolation from the rest of the system components by targeting immobilized subjects under CO_2_ anesthesia. This was done for several reasons: targeting immobilized subjects is a much simpler endeavor than for flying subjects, which allows mortality testing earlier in the development process and permits higher throughput testing; it establishes a baseline for the feasibility of killing or disabling insects with optical energy separately from the performance of the rest of the system; and it helps lead to the identification of the best candidate lasers for the full system to greatly reduce the testing burden for future in-flight studies. The goal of this study was thus to determine the lethality of multiple lasers at various wavelengths, spot sizes, powers, and pulse durations on anesthetized female *Anopheles stephensi* mosquitoes. This species was chosen as a representative organism due to its size, its robustness in a laboratory setting, and its importance as a malaria vector. Multiple parameters were explored for each laser, with the parameter (and laser) selection guided by potential requirements and practical considerations for the future deployable system (e.g. relatively inexpensive, robust, good beam quality, etc.). Even with these guidelines, there were numerous potential values to investigate for each parameter; in general, we chose to investigate a coarse sample of these values to obtain a broad baseline performance. The results obtained from this study provided clear indications of which optical parameters had the most significant influence on mosquito mortality, and therefore which lasers would be most practically deployed.

## Results

### Dosing with millisecond-scale pulses at 532nm

**Figure 1:**
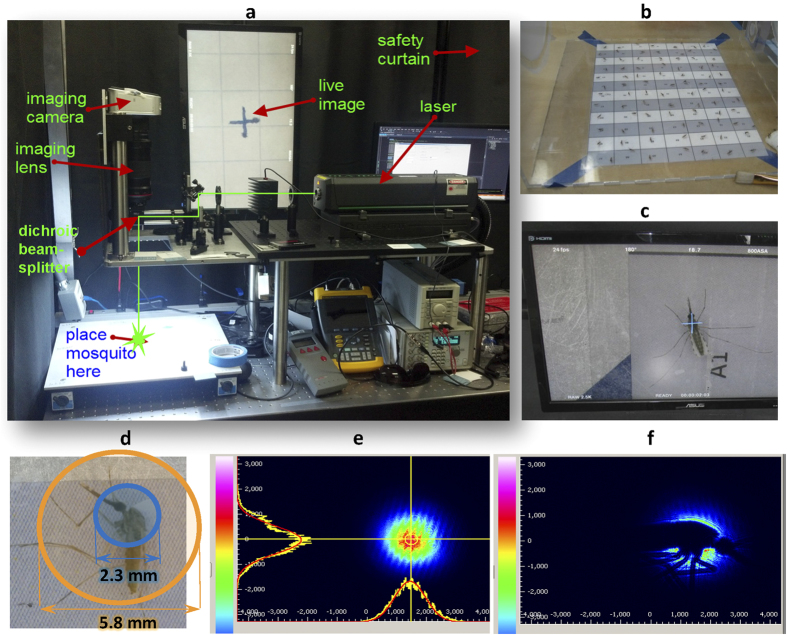
Dosing system setup and mosquito targeting. (**a**) The current dosing laser (here, Coherent Verdi V10) was axially co-aligned with an imaging camera (Blackmagic Cinema Camera EF) using a dichroic beamsplitter that reflected the laser light down to the test subject stand and passed other visible light (provided by LED strips on bottom of optical platform) from the subject stand up through a telescopic macro lens (Canon EF 180mm f/3.5 Macro) and into the camera. Live images from the camera were displayed on a monitor above the setup. The two optical paths projected through an opening in the optical platform onto the (**b**) floor of a test cage, where anesthetized mosquitoes were arranged in a 12 by 7 grid. The distance from the laser to the subject was approximately two meters. (**c**) The view from the camera with a crosshair noting the location and extent of the co-aligned laser was used to accurately target the mid-thorax of one subject at a time. (**d**) Circles indicate which areas of the mosquito were hit by the two typical beam diameters. (**e,f**) Images from CCD beam profiler of 2.3 mm diameter, 532 nm wavelength spot (**e**) without and (**f**) with a subject present validated targeting accuracy and showed that the most intense portions of the Gaussian beam impacted the mosquito. Note that the interference fringes in (**e**) are from the presence of a glass slide near the sensor to facilitate mosquito placement.

Dosing experiments were first carried out at 532 nm using the setup shown in Fig. 1 (see Supplementary Video S1 for a demonstration and Supplementary Methods for complete laser and optics details). The optical beam was roughly centered on the mosquitoes’ thoraces (Fig. 1c–f), as this allowed the beam to hit many key mosquito structures; it also coincides with the approximate location of the body centroid during flight, which will come into play in future in-flight studies. For each laser condition in a given test (full list below in Table 1), the primary outcome was the mortality rate of a sample of 84 female *An. stephensi* subjects 24 hours after exposure. As defined by the World Health Organization (WHO), mortality rate included mosquitoes that were dead and those that were moribund – i.e. could not stand or fly properly^34^ (see Supplementary Video S2 for a demonstration of determining health status). From a functional perspective, creating moribund mosquitoes in the wild is equivalent to killing them. Dose-response curves were then created by performing a logistic regression fit for mortality rate as a function of optical fluence (J/cm^2^). As the optical pulse energy per unit area, fluence can be varied by changing either the beam spot size or the pulse energy, which is the product of optical power and pulse duration. Since fluence can capture changes in any one of the experimentally controlled values (power, pulse duration, spot size), it was deemed the best parameter to plot mortality against throughout the experiments.

**Figure 2:**
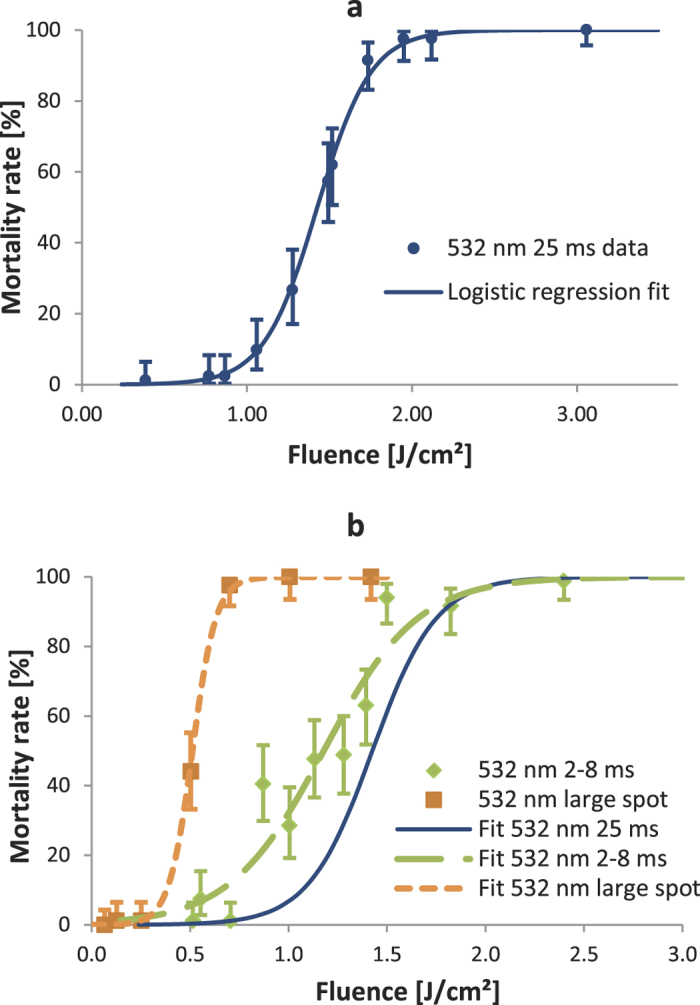
Mortality data and dose-response curves for 532 nm millisecond scale dosing. (**a**) Data points each represent mortality outcomes from dosing 84 female *An. stephensi* with 532 nm wavelength, 2.3 mm Gaussian beam diameter, and 25 ms pulses, with variable power 0.5 to 4 W. Error bars are plus and minus exact binomial 95% confidence intervals. Curve fit achieved via logistic regression with ordinary R^2^ = 0.99 and LLR pseudo R^2^ = 0.96. (**b**) Solid blue line is fit from (a) for comparison. Green diamonds and long-dashed line are data and logistic regression fit (ordinary R^2^ = 0.92 and LLR pseudo R^2^ = 0.82) for 532 nm, 2.0 mm Gaussian beam, 8.5 W power, and variable 2 to 8 ms pulses. Orange squares and short-dashed line are data and logistic regression fit (ordinary R^2^ = 0.99 and LLR pseudo R^2^ = 0.98) for 532 nm, 5.8 mm Gaussian beam, 8.5 W power, and variable 2 to 45 ms pulses. For both, error bars are exact 95% binomial confidence intervals.

Figure 2a shows the dose-response curve for 532 nm wavelength, 2.3 mm Gaussian beam diameter, and 25 ms pulse length. Pulse energy, and therefore fluence, was altered by adjusting the laser power from 0.5 to 4 W. The data points produced a prototypical sigmoidal curve, as the logistic regression procedure fit the data with an ordinary R^2^ = 0.99 and the commonly used log-likelihood ratio (LLR) pseudo R^2^ = 0.96. This tight curve fit, along with a negative (un-dosed) control mortality of only 1.5%, indicated that the dosing system and procedure were highly repeatable and robust to any variability besides the fluence delivered to the mosquito. The lethal dose 90% (LD90) value of 1.78 J/cm^2^ corresponded to just under 3 W of optical power at the 25 ms pulse duration, which is easily achieved by several laser technologies in the blue to green wavelength spectrum.

Other tests at 532 nm examined the effects of higher power, shorter pulses, and of a larger beam diameter that encompassed nearly the entire mosquito. Figure 2b shows these other dose-response curves relative to the fit from Fig. 2a. Reducing pulse durations from 25 ms (Fig. 2a) to 2 to 8 ms and using a higher constant power setting of 8.5W (instead of 0.5 to 4 W) did not substantially reduce the fluence value required to obtain high mosquito mortality (green, long-dash line in Fig. 2b). With the power still at 8.5 W, the beam was expanded to 5.8 mm to examine whether it is advantageous to hit the whole subject versus only a portion of it (Fig. 1d). As seen in the short-dash, orange line in Fig. 2b, this condition had an LD90 of 0.64 J/cm^2^, which corresponded to a 20 ms pulse. Comparing this result to both other curves in Fig. 2b, the large spot LD90 was ~3× lower fluence, but over a ~6× larger spot, meaning that the net pulse energy requirement was greater than that for the smaller spot sizes.

**Figure 3:**
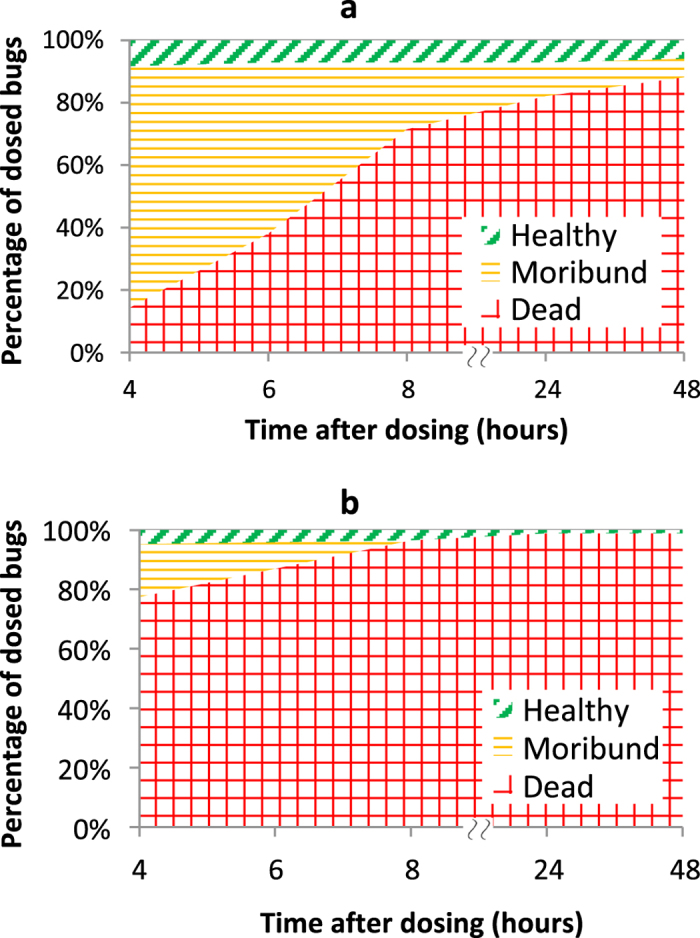
Time course of mortality following 532 nm dosing. Percentages of dosed subjects that were alive (green diagonal hatching), moribund (orange horizontal hatching), and dead (red cross hatching) at set intervals following the dose. Note the discontinuity in the x axis between 8 and 24 hours. There were 84 subjects each for (**a**) LD90 condition from the 2.3 mm, 25 ms curve and (**b**) LD90 condition from the 2.0 mm, 2–8 ms curve. Negative control showed greater than 95% healthy mosquitoes at all time points.

To investigate how quickly mosquitoes were disabled, a time course study of mortality was also performed with single cages of 84 subjects at the LD90 levels for the 25 ms, 0.5–4 W and 8.5 W, 2–8 ms conditions from above. Figure 3 shows the breakdown among healthy, moribund, and dead subjects at 4, 6, 8, 24, and 48 hours following exposure. At times less than four hours, lasting effects of the anesthesia could be mistaken for moribundity. In general, if a subject appeared healthy at four hours, it remained that way through 24 and even 48 hours. In the higher power condition (8.5 W for 5.7 ms; Fig. 3b), the non-surviving mosquitoes largely died immediately or over the first several hours following exposure. In the lower power condition (3 W for 25 ms; Fig. 3a), few mosquitoes died right away, and the largest fraction (75%) emerged from the anesthesia in a moribund state. These moribund mosquitoes then proceeded to die off over the remainder of the 24 hour period. The likely explanation for these differing trends is demonstrated in Supplementary Videos S3 and S4. In videos recorded at 2000 frames per second, the lower power condition (Supplementary Video S3) caused relatively little reaction by the mosquito or obvious outward signs of physical damage. In Supplementary Video S4, however, the higher power condition created a small plume of smoke. Thus, the higher power condition appeared to create a more severe thermal injury that led to immediate death, while the lower power condition wounded them such that they could not survive for any significant amount of time. From a functional perspective, these outcomes were largely equivalent in quickly eliminating the presence of healthy insects that could theoretically spread disease or cause crop damage; for both conditions, the total dead/moribund counts increased by less than 5% from the initial four hour count to the official 24 hour count. Formal time-course studies were not carried out for the additional lasers below, but the trends identified here appeared to persist with little variation.

### Dosing with Q-switched lasers

**Figure 4:**
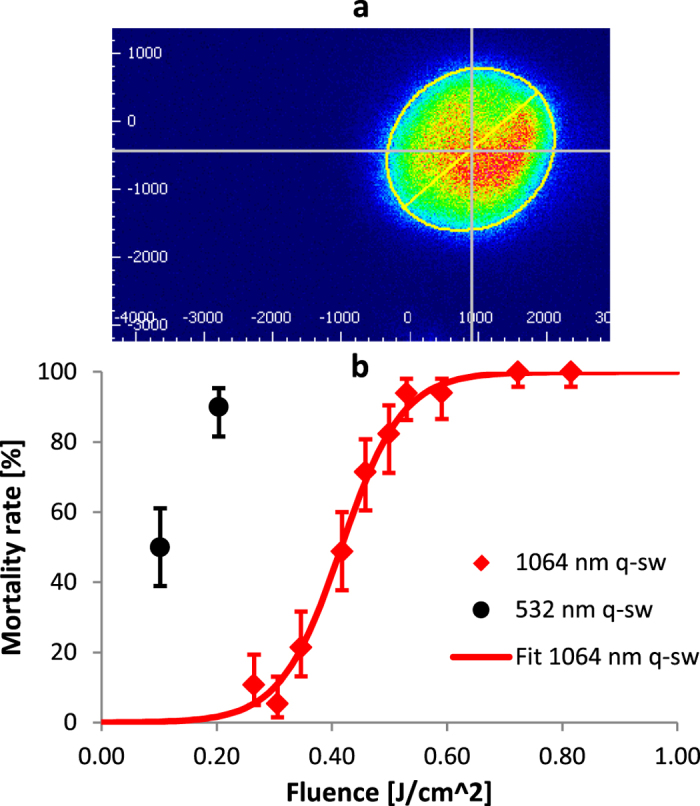
Dosing outcomes from Q-switched Nd:YAG laser. (**a**) Representative beam profile of Q-switched Nd:YAG laser. (**b**) Mortality from dosing with 2.5 mm diameter, 10 ns pulses with 5 mJ and 10 mJ pulse energies at 532 nm (black circles) and with variable 12 to 40 mJ pulse energies at 1064 nm (red diamonds). For both, error bars are exact 95% binomial confidence intervals. Red solid line is logistic fit for 1064 nm data (ordinary R^2^ = 0.99 and LLR pseudo R^2^ = 0.92).

For a deployable insect killing system as described above, using optical pulses longer than tens of milliseconds is not practical, so that regime was not investigated here. In theory, pulses much shorter than tens of milliseconds could be advantageous if they were to lead to much lower lethal energy requirements, or if they eliminated the need to track the mosquito while dosing it (i.e. if the mosquito cannot move any appreciable distance relative to the beam diameter, the laser can simply be fired rather than steered to stay on the target during dosing). To investigate this possibility, a Q-switched neodymium-doped yttrium aluminum garnet (Nd:YAG) laser that provided 10 ns pulses with varying energy at both 532 nm and 1064 nm was used. In both cases, the beam diameters were 2.5 mm, with a profile in between uniform and Gaussian (Fig. 4a), as is typical for this type of laser. Figure 4b shows a full dose-response curve at 1064 nm and a limited data set for 532 nm; the data set was limited to two points due to difficulties achieving a stable output at low enough energies. At 532 nm, the LD90 was around 10 mJ, or 0.25 J/cm^2^. Although this was a ~7× reduction in lethal fluence compared with the millisecond-scale dosing, the peak power for these pulses was dramatically larger at 1 MW. At these conditions, interactions between the laser pulse and biological tissues are referred to as “stress confined” (also see Supplementary Discussion 1), in which mechanical damage can extend beyond any thermal damage^35^. Supplementary Video S5 demonstrates this difference versus Supplementary Videos S3-S4 from lower power dosing. The downside is that these high power interactions also present challenges regarding damage to optical components and to safety, as discussed below.

The Nd:YAG laser was also operated in its more stable 1064 nm configuration (all other optical parameters equivalent) to investigate differences between visible and near-infrared wavelengths. For an organism whose pigmentation is dominated by melanin, as is the case for most mosquitoes and numerous other insects^36^, the absorption coefficient for 1064 nm light is several times lower than for visible light in the blue to green range^37^. As seen in Fig. 4b, this absorption difference led to an increase in LD90 fluence from 0.25 J/cm^2^ at 532 nm to 0.53 J/cm^2^ at 1064 nm, which corresponded to 26 mJ at the 2.5 mm beam diameter.

### Using longer pulses around 1 μm wavelength

**Figure 5:**
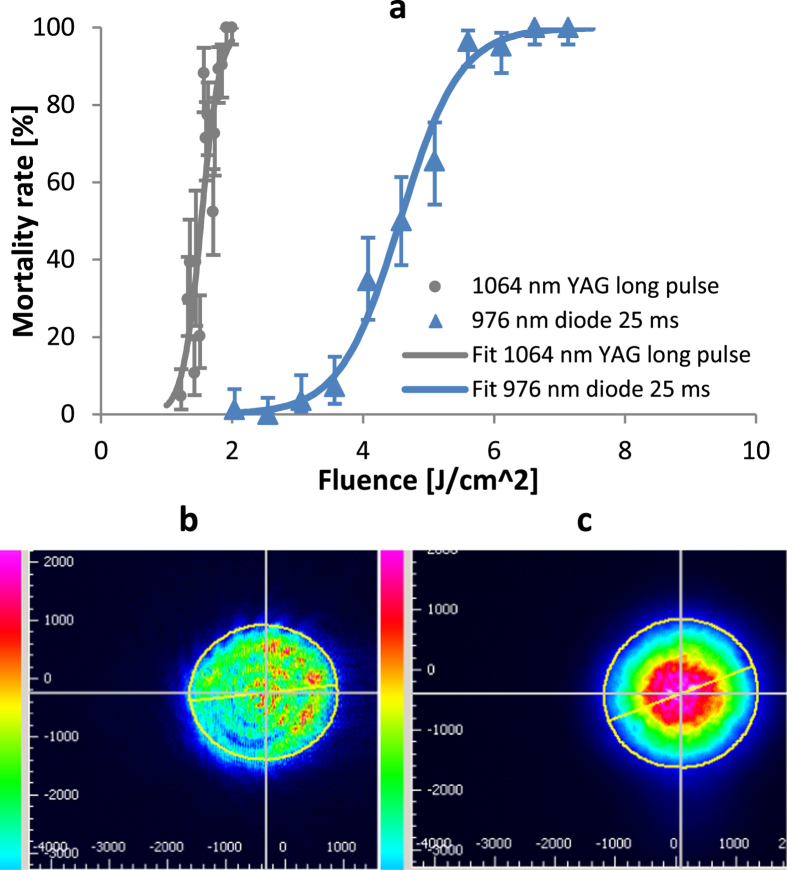
Dosing with lower power 1 μm sources. (**a**) Gray circles and solid line are mortality data and logistic regression fit (ordinary R^2^ = 0.8 and LLR pseudo R^2^ = 0.68) for dosing with the Nd:YAG laser in “long pulse” mode, producing 2.5 mm diameter spots, 90 μs pulses, and variable pulse energies of 60–100 mJ. Light blue triangles and dashed line represent data and logistic regression fit (ordinary R^2^ = 0.99 and LLR pseudo R^2^ = 0.94) for dosing with 976 nm diode making 2.5 mm diameter spots and 25 ms pulses with variable power 4–14 W. (**b**) Representative beam profile from Nd:YAG laser in “long pulse” mode, showing typical spikes and banding. (**c**) Representative beam profile from 976 nm diode showing round shape and Gaussian intensity profile.

Numerous types of laser sources operating near 1 μm wavelength are widely available and relatively cheap. Since the shift in LD90 from 532 nm to 1064 nm with Q-switched pulses was only ~2×, we chose to investigate other sources near 1 μm with lower powers and longer pulses as well. Figure 5a shows the dose-response curves for the same Nd:YAG laser operating in “long-pulse” mode (90 μs pulse duration) at 1064 nm and for a 976 nm diode laser providing 25 ms pulses. Both lasers used 2.5 mm diameters, although the Nd:YAG provided a jagged, flat top-like profile (Fig. 5b), and the diode provided a largely Gaussian profile (Fig. 5c). Given the optical absorption properties of melanin and other biological molecules^37^, it was expected that the wavelength difference between 976 nm and 1064 nm would have a negligible impact on the mortality results.

The “long-pulse” Nd:YAG study resulted in an LD90 of 1.84 J/cm^2^, which was ~3.5× greater than for the Q-switched pulses at the same wavelength and nearly equivalent to the millisecond scale 532 nm results. Given the other optical parameters, this LD90 condition was 90 mJ and 1 kW. The data showed more variability than other tests and had the worst logistic regression fit (ordinary R^2^ = 0.8, adjusted R^2^ = 0.68), likely due to the inconsistent nature of the jagged beam profile. With 25 ms pulses from the much lower power 976 nm diode (4 to 14 W tested), the LD90 further increased to 5.57 J/cm^2^, or 273 mJ at ~11 W. The curve fit returned to its typically good outcome, with ordinary R^2^ = 0.99 and adjusted R^2^ = 0.94, likely due to the more consistent beam profile of this laser (Fig. 5c). Although the LD90 fluence and pulse energy increased by an order of magnitude from the Q-switched Nd:YAG to the diode laser, the required power decreased by five orders of magnitude.

### Targeting water absorption with infrared lasers

**Figure 6:**
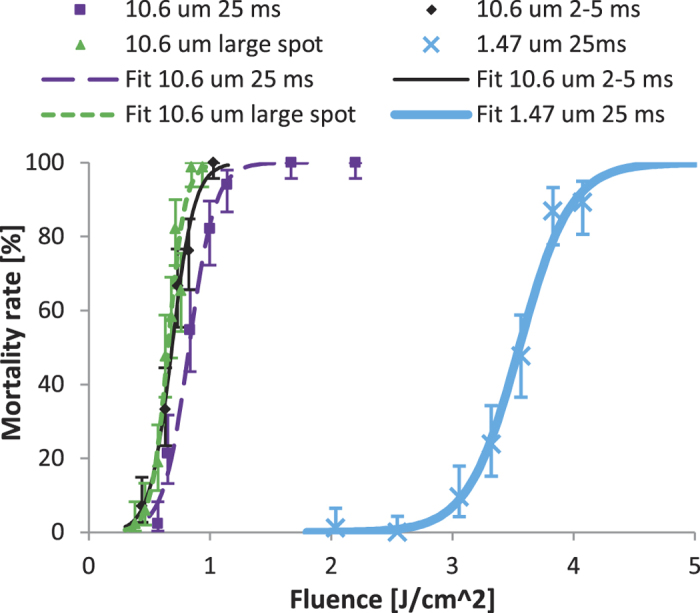
Mortality from longer wavelength infrared sources. CO_2_ laser (10.6 μm wavelength) mortality data and logistic regression fits from 2.5 mm Gaussian beam, 25 ms pulses, variable power 1–3 W (purple squares and long-dashed line, fit with ordinary R^2^ = 0.99 and LLR pseudo R^2^ = 0.93); from 2.5 mm beam, 10 W, variable pulse length 2–5 ms (black diamonds and thin solid line, fit with ordinary R^2^ = 0.99 and LLR pseudo R^2^ = 0.90); and from 5.8 mm Gaussian beam, 25 ms pulses, variable power 2–10 W (green triangles and short-dashed line, fit with ordinary R^2^ = 0.96 and LLR pseudo R^2^ = 0.88). Blue X’s and thick solid line are data and fit (ordinary R^2^ = 0.99 and LLR pseudo R^2^ = 0.94) from 1.47 μm diode producing 2.5 mm Gaussian beam, 25 ms pulses, variable power 4 to 10 W. In all cases, error bars are plus and minus exact binomial 95% confidence intervals.

Light in the blue to green spectrum is strongly absorbed by various pigments and oxygen carrying molecules, while light near 1 μm is weakly absorbed by the same molecules and also weakly absorbed by water. Further into the infrared spectrum around 1.5 μm and 10.6 μm wavelengths, optical absorption is dominated by water. Absorption by water is moderate near 1.5 μm and very strong at 10.6 μm^38^. Laser sources at these wavelengths are also plentiful and can be inexpensive, as the 1.5 μm region comprises multiple telecom bands, and CO_2_ lasers emit around 10.6 μm. Here, we tested a 1.47 μm diode laser and a 10.6 μm CO_2_ laser to examine how responses to mid- and far-infrared lasers may differ from visible and near-infrared lasers. For the diode laser, a single test was performed with a 2.5 mm diameter, mostly Gaussian beam and 25 ms pulses with variable power (4 to 10 W). As seen in Fig. 6, this test produced an LD90 of 4.02 J/cm^2^, or just under 8 W for the 25 ms pulse. As expected, this value was lower than for the 976 nm diode 25 ms pulses, but still several times greater than for the visible lasers.

Three different tests were performed with the CO_2_ laser. As Fig. 6 shows, the typical 2.5 mm Gaussian beam and 25 ms pulse test had an LD90 of 1.06 J/cm^2^, which equates to 2.1 W. With the power set to 10 W and pulse duration varied from 2 to 5 ms, the LD90 showed a modest decline to 0.89 J/cm^2^, or 44 mJ over 4.3 ms. Similar to what was done with the 532 nm laser, the beam was also expanded to 5.8 mm diameter to cover the majority of the subject cross section. With variable power over 25 ms, the LD90 came out at 0.80 J/cm^2^; this corresponds to 211 mJ and 8.4 W. Thus, unlike at 532 nm, the required lethal fluence for the CO_2_ laser did not decrease significantly from the smaller spot to the larger spot.

### Determining blood feeding rate compared with mortality rate

**Figure 7:**
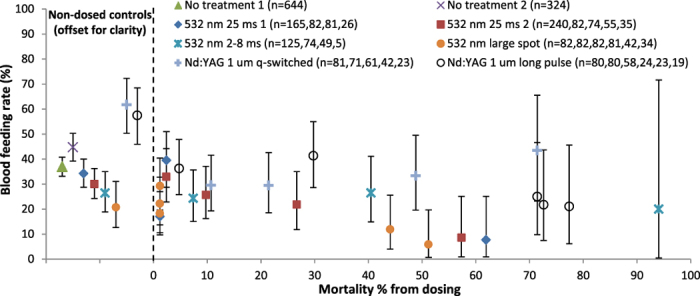
Blood feeding versus dosing mortality. Percentages of subjects that took a sheep’s blood meal from an artificial feeder are plotted as a function of survival from a previous dosing experiment. Subjects that underwent no experimental treatment or that were negative dosing controls are shown offset to the left of 0% mortality for clarity. Legend lists numbers of subjects offered the blood meal for each point left to right in a given series (starting with control). Error bars are plus and minus exact binomial 95% confidence intervals.

Besides creating moribund mosquitoes, another lower power “functional kill” outcome of dosing could be mosquitoes that appear healthy but are impaired such that they cannot take a blood meal, thus making them unable to spread disease. To investigate this possibility, we offered sheep’s blood (a standard alternative to human blood) to the surviving mosquitoes from a subset of the above dosing experiments. The data offset to the left side of Fig. 7 shows the blood feeding rates for untreated subjects (i.e. never left the insectary) and negative dosing controls. These rates were highly variable, so establishing a true baseline proved impossible. Within a given series, the presence of a significant correlation between mortality rate and blood feeding rate was inconsistent as well. For example, the “532 nm 25 ms 2” data showed a significant negative correlation (Pearson’s r = −0.98, *p* value = 0.0028), but the “532 nm 2–8 ms” data showed no significant correlation (*p* value = 0.20). Given the lack of consistent, meaningful results obtained from this complex biological process, blood feeding was abandoned as an additional outcome metric and was not performed for tests conducted after those included in Fig. 7.

## Discussion

The results presented here, as summarized in Table 1, provide several implications for the use of lasers to kill small flying insects. The lethal fluence required at different wavelengths, all other parameters being equal, strongly correlates with the mosquitoes’ optical absorption properties. This finding makes sense because for the same pulse conditions, a higher absorption coefficient will lead to a higher peak temperature achieved in the subject (more efficient conversion of optical energy to heat), thus causing more severe thermal damage. With *An. stephensi*, the LD90 for 532 nm was significantly lower than for 1064 nm, since the former is absorbed much stronger by mosquito pigments and oxygen-carrying molecules, and the LD90 for 10.6 μm was lower than for 1.47 μm, as the former is absorbed several times stronger by water. It is more difficult to compare the visible and near infrared results with the mid- and far infrared results; since they target different optical absorbers (pigments and oxygen carrying molecules versus water, respectively), the optical energy is deposited with different spatial distributions, according to where the absorbers are distributed in the subject. Given this dependence on spatial distributions of optical absorbers, the wavelength findings presented here for *Anopheles* mosquitoes, particularly for the visible to near infrared range, could be sensitive to factors like which pigments are present, cuticle thickness, and other structural properties among different target organisms.

A more consistent trend is that for the same wavelength and beam diameter, shorter pulses led to lower lethal fluence values. The relative magnitudes of the fluence reductions versus pulse durations likely depended on how close a given set of conditions was to stress confinement, thermal confinement, or no confinement^35^ within the area of the mosquito hit by the laser pulse. A full description of confinement zones is provided in Supplementary Discussion S1 and Supplementary Figure S1. In short, for this application these zones describe combinations of pulse duration and of optical absorption by the mosquito that lead to inefficient heating of the laser irradiated area due to heat leaking into surrounding tissue (no confinement), efficient heating of just the laser irradiated area (thermal confinement), or efficient heating of the laser irradiated area plus mechanical disruption in the irradiated and immediately surrounding area (stress confinement). Depending on wavelength, it was likely that the typical 25 ms pulses were in thermal confinement, even if they were near the border of thermal and no confinement. By reducing the pulse length to a few milliseconds, there was no appreciable movement between zones, so the required fluence levels did not change dramatically. More substantial reductions in required fluence were seen with the Q-switched laser, as 10 ns pulses lay in the stress confinement region, in which both thermal and mechanical damage are seen.

The above discussion focuses on changes in lethal fluence. As noted previously, fluence was deemed the most appropriate independent variable to analyze across the experiments, but it cannot be examined in isolation. Although the Q-switched pulses allowed for the lowest fluence kills, the peak powers were several orders of magnitude greater than for other tests. Thus, such a laser would likely only be a viable solution in very specific cases. Since 25 ms pulse dose-response curves and single digit ms pulse dose-response curves had similar LD90 fluence values, it would be hard to justify the use of a laser with 4–5× the power to use a 5 ms pulse compared with a lower power 25 ms pulse without further system requirements driving the choice. Similarly, the larger spot experiments demonstrated a net increase in pulse energy, and therefore power required relative to the smaller spots. Laser cost and safety concerns scale nearly linearly with power, so there are multiple factors pushing against the use of higher power lasers. For safety in particular, the maximum permissible exposure (MPE) to a laser pulse, given in terms of fluence, decreases as pulse duration decreases, and therefore as power increases. This directly impacts the width of the “no-fire zone” along the length of the PF active area noted above where the system would shut down if it detected people or large animals present. For 25 ms pulses, the infrared wavelengths (which do not reach the retina) would require exclusion zones as small as less than a meter. Shorter wavelengths and pulse durations both lead to larger exclusion zones. Beam diameters less than 2 mm might have reduced the power requirement even further, especially since a greater percentage of the beam area would be incident on the sample. We do not anticipate being able to produce spots of that size in a deployable system without using large (and therefore expensive) optical elements, though, so no studies were performed.

These experiments demonstrated that *An. stephensi* can be killed or disabled by optical pulses under several different conditions at various wavelengths. More importantly, these results can be generalized to the use of relatively cheap, robust lasers with sufficient beam quality to be focused over a long range. Subsequent work will test the most favorable conditions identified here on subjects that are freely flying within test cages rather than lying immobilized on the cage floors, and will attempt to confirm our preliminary indications that such “in-flight dosing” does not require substantially greater lethal energies than the anesthetized dosing did. We will then expand the studies to other representative species as well.

## Methods

### Mosquitoes

The mosquitoes used in this study were *Anopheles stephensi* clone STE 2, originally from the Malaria Research and Reference Reagent Resource Center (MR4), provided by the Center for Infectious Disease Research (CIDR). Females were separated from males prior to transfer to the IV Lab. In the IV Lab insectary, which was maintained at 28 °C and 80% relative humidity, the adults were fed a solution of 10% dextrose in water via a soaked cotton ball. At least 24 hours prior to laser exposure, the subjects were mouth aspirated from the CIDR-supplied transfer cages into our test cages, with approximately 120 individuals per test cage. The test cages were 11′′ cubes constructed from polycarbonate, with polyester mesh covering six inch diameter openings on three faces. Each test cage had a removable top and a hose barb for delivery of CO_2_ (through another small mesh). Dosing experiments were conducted four to five days after shipment to IV Lab, such that our subjects were seven to ten days old (post-emergence) at the time of the dosing experiments. This age is ideal for *Anopheles* because in the wild, they have had or are seeking their first blood meals at this time, but they cannot yet transfer malaria due to the time it takes the *Plasmodium* to reproduce in the mosquito.

### Dosing System Operation

The configuration of the dosing system was described by Fig. 1. Its operation was controlled by a simple, custom program running on a personal computer. When the program received the signal from a foot pedal, it commanded the mounted video camera to begin recording video, paused briefly (~300 ms), then sent a trigger to a digital delay/pulse generator. The pulse generator controlled the timing and duration of the laser pulse, either by signaling an electronic fast shutter or by sending a pulse to a laser driver, depending on which laser was currently in use. After two more seconds, the program commanded the camera to stop recording. The camera recorded its video to an onboard solid state disk drive; these videos were archived on a file server, available in the event there was any need to review dosing imagery.

### Dosing Experimental Protocol

On the day of an experiment, test cages were transferred by vehicle or cart from the insectary to the test lab, which was in a separate building at IV Lab. The test lab was cooler and less humid than the insectary, but initial control experiments showed no statistically observable mortality associated with up to eight hour visits to the test lab. Beam profiles were taken at least at the beginning of each day’s experiments, and pulse energy was measured several times before and after each test cage.

When a cage was to be dosed (or handled as a non-dosing control), the dextrose-soaked cotton ball was removed, the nylon meshes were covered with plastic lids, and a flow of pure CO_2_ was delivered into the cage at a flow rate of 50 cubic feet per hour for two minutes via a flexible tube. Generally all of the subjects were unconscious after two minutes. The flow rate was then reduced to 15 cubic feet per hour for the duration of the dosing experiment, which lasted 15–20 minutes per cage. Once all the subjects were sedated, the lid of the test cage was removed. Out of the approximately 120 subjects in the cage, 84 apparently healthy female mosquitoes were selected and sorted into a seven by twelve grid on the floor of the cage (see Fig. 1b). A soft horsehair brush was used to handle the subjects, and care was taken when sweeping the brush to avoid any downward force. Extra subjects (including all males that may have been in the cage) were removed and discarded. The sorted cage was moved to the dosing apparatus while maintaining the flow of CO_2_. By manually sliding the cage on its stand, all 84 subjects were presented to the laser/camera in sequence, with a single laser pulse targeted and administered as described above. Largely through chance from the outcome of the handling during the sorting, many subjects presented the lateral side of their thorax to the laser; others the ventral thorax, and to a lesser degree the dorsal side of the thorax. When all of the subjects were dosed, the lid was restored, the CO_2_ delivery was halted, the plastic covers were removed, and the cotton ball with dextrose solution was returned to the cage mesh. The total dosing time of each cage was noted.

Non-dosed controls were handled in the same fashion, except for the laser dosing itself. The 84 mosquitoes were sorted onto the grid, and the CO_2_ was administered for the maximum typical duration of the dosing (20 minutes). After this interval, the CO_2_ flow was halted and the cages restored in the same fashion as for the dosing experiments. One handling control cage was processed during each day of testing. We also conducted a mortality control on each day of tests, once an LD50 was determined for a given set of conditions. The mortality controls were conducted like any other dosing test; simply a dosing of 84 subjects at the defined LD50 fluence to ensure that each batch of mosquitoes was similarly robust. After conducting all of a given day’s tests and controls in the test lab, the cages were returned to the insectary.

### Mortality Counts

Mortality counts were conducted in accordance with the WHO guidelines for evaluating mosquito adulticides^34^. At 24 hours (plus or minus two hours) after the dosing experiment, the number of subjects that appeared healthy and the number that appeared dead or moribund (i.e. alive, but unable to fly properly) were recorded for each cage (demonstration in Supplementary Video 2). For one set of tests, as a time study for mortality and moribundity, both mortality and moribundity counts were conducted after 4, 6, 8, 24, and 48 hours.

### Data Analysis

Mortality data was analyzed as a function of fluence (J/cm^2^). For a given test, the fluence was altered by changing the power with a constant pulse duration (thus changing pulse energy) and spot size, or by changing the pulse duration with a constant power (again changing pulse energy) and spot size. Spot size was only changed between tests, and was always constant for a given dose-response curve. Error bars were constructed for each data point by determining the exact 95% confidence intervals of the relevant binomial distribution (i.e. *p* deaths/disablements out of *n* trials in a single cage)^39^. For each set of tests on a given optical variable, a dose-response curve was then created by performing logistic regression on mortality as a function of fluence. The regression was performed via MATLAB’s built-in generalized linear model framework with a logit link function, which also provided R^2^ and pseudo-R^2^ values (more widely used than ordinary R^2^ in logistic regression) to assess the model’s goodness of fit. The LD50 and LD90 fluence values were determined from the dose-response curve, and then used as benchmarks for comparing the effectiveness of the various optical configurations.

The choice of 84 subjects per test cage stemmed from an initial sample size analysis with the software program GPower. We wanted to ensure that a point at 90% mortality would have a 95% confidence interval of no larger than plus or minus 10% mortality, assuming the common power analysis values of α = 0.05 and power = 0.8. The choice of number of data points per curve was more arbitrary, and represented a compromise between experimental throughput and ensuring coverage across the range of mortality outcomes.

### Blood Feeding Protocol

For the first several experiments, assuming a sufficient number of subjects (five or more) survived a given test, a follow-on test was conducted on the remaining subjects to measure their propensity to blood feed. After the 24 hour mortality count, to establish fasting conditions, the feeding cotton ball with dextrose solution was replaced by a cotton ball containing only water. At 24 hours later, a meal of fresh sheep’s blood was provided to the test cages. The feeders were bell shaped jars (Lillie Glassblowers) with a water jacket for maintaining the blood at 37–°C throughout the test. A collagen membrane was stretched in a watertight fashion across the open bottom of the bell. The feeders were placed on top of a nylon mesh of each cage to feed. When the cages were ready, 10 ml of warm sheep blood were added. The mosquitoes were allowed 30 minutes to feed through the mesh and the collagen membrane. After 30 minutes, the feeders were removed. A count was conducted of engorged subjects (fully swollen abdomens). Control test cages were blood fed alongside dosing test subjects, in identical fashion. Approximately halfway through the course of these dosing experiments, the follow-on blood feeding experiments were discontinued, as described in Results.

## Additional Information

**How to cite this article**: Keller, M. D. *et al*. Laser induced mortality of *Anopheles stephensi* mosquitoes. *Sci. Rep.* **6**, 20936; doi: 10.1038/srep20936 (2016).

## Supplementary Material

Supplementary Information

Supplementary Video S1

Supplementary Video S2

Supplementary Video S3

Supplementary Video S4

Supplementary Video S5

## Figures and Tables

**Table 1:** Summary of dosing experimental parameters and LD90 conditions.

Laser experimental parameters				LD90 Conditions			
Wavelength (nm)	Beam Diameter (mm)	Pulse duration (ms)	Power (W)	Fluence (J/cm^2^)	Pulse duration (ms)	Pulse energy (mJ)	Power (W)
532	2.3	25	(0.5–4)	1.8	25	74	3.0
532	2.0	(2–8)	8.5	1.7	5.7	48	8.5
532	5.8	(2–45)	8.5	0.6	20	170	8.5
532	2.5	1e-5	(0.5–1.0)e + 6	0.3	1e-5	10	1.0e + 6
1064	2.5	1e-5	(1.3–4.0)e + 6	0.5	1e-5	26	2.6e + 6
1064	2.5	0.09	(0.7–1.1)e + 3	1.8	0.09	90	1.0e + 3
976	2.5	25	(4–14)	5.6	25	273	10.9
10,600	2.5	25	(1.1–3.3)	1.1	25	52	2.1
10,600	2.5	(2–5)	10.2	0.9	4.3	44	10.2
10,600	5.8	25	(2–10)	0.8	25	211	8.4
1470	2.5	25	(4–10)	4.0	25	197	7.9
