# Supplementary material for: Laser induced mortality of Anopheles stephensi mosquitoes
Source: Sci Rep. 2016 Feb 18;6:20936. doi: 10.1038/srep20936 (PMC4758184; doi:10.1038/srep20936)

## Supplementary Information for "Laser induced mortality of *Anopheles stephensi* mosquitoes" by Matthew D. Keller, David J. Leahy, Bryan J. Norton, 3ric Johanson, Emma R. Mullen, Maclen Marvit, Arty Makagon

### Supplementary Video Captions

**Supplementary Video S1 – Dosing demonstration from video camera's perspective.** View is from the video camera used for aligning the bug with the laser (see Fig. 1) during dosing. Recordings from the first 14 bugs are stitched together, starting 300 ms prior to the laser pulse and extending 2 seconds past the laser pulse for each dose. Dosing conditions were 532 nm, 2.3 mm spot, 8.5 W, 25 ms pulse duration, which was more than sufficient (~ 4x the LD90 fluence) to kill every subject. Several can be seen severely twitching following the pulses, and a spot of condensation can be seen forming and quickly disappearing behind many of the subjects. This condensation was confirmed to come from the subject. For less lethal conditions, the twitching and condensation were less severe or not present.

**Supplementary Video S2 – Determining mortality results.** Video shows how dosing cages look during mortality counts; here, for a box with ~50% mortality. Initial pan shows several surviving bugs resting on the walls, and other subjects on the ground. Subsequent video shows how the health of bugs on the ground is determined, first by shaking the box and blowing on the subjects, then by tapping the bottom of the cage. In the "shaking and blowing" section, several subjects can be seen getting up and flying normally, indicating their healthy status. Others remain unmoving (dead) or demonstrate a moribund status by spinning on the ground or flying in an uncoordinated manner into the back wall. Similar results are seen in the "tapping" section.

**Supplementary Video S3 – High speed video of lowest power 532 nm dosing.** Close-up video of subject being dosed with 532 nm, 3 W, 2.5 mm beam, for 25 ms; this was the LD90 condition for the curve with 532 nm, 2.5 mm beam, constant 25 ms pulse, and variable power. Video was recorded at 2000 frames per second and played back at 1/67x speed. There is relatively little reaction by the bug other than a small twitch, and no easily visible signs of damage.

**Supplementary Video S4 – High speed video of highest power 532 nm dosing.** Close-up video of subject being dosed with 532 nm, 8.5 W, 2.0 mm beam, for 5.7 ms; this was the LD90 condition for the curve with 532 nm, 2.0 mm beam, constant 8.5 W, and variable pulse duration. Video was recorded at 2000 frames per second and played back at 1/67x speed. A small plume of smoke is a clear indication that a serious thermal injury was imparted on this subject.

**Supplementary Video S5 – High speed video of Q-switched 532 nm dosing.** Close-up video of subject being dosed with 532 nm, 2.5 mm beam, 10 ns pulse, and 10 mJ pulse energy, or 1 MW power; this was the approximate LD90 condition for the test with 532 nm, 2.5 mm beam, constant 10 ns pulse, and variable pulse energy. Video was recorded at 180,000 frames per second and played back at 1/3600x speed. The variable brightness is due to the use of a strobe flash to produce enough light for use at this extremely high frame rate. Note the material ejection and momentum imparted to the upper thorax and head of the subject.

**Supplementary Methods – Details of lasers and optics used in the experiments.**

In the work reported here, five lasers were used: a diode-pumped solid state laser (Coherent Verdi V10) operating at 532 nm; a Q-switched Nd:YAG laser (Spectra-Physics GCR-250) operated at both 532 nm and 1064 nm; a 976 nm diode laser (IPG Photonics, PLD-33-A-976); a 1470 nm diode laser (QPC Lasers 6014-M002); and a 10.6 μm $CO_2$ laser (Synrad Firestar V30). Each will be discussed briefly in turn.

The Coherent Verdi V10 is a 10 W continuous wave (CW) laser operating at 532 nm. Most of the experiments conducted with this laser used the highly collimated 2.0 - 2.5 mm diameter output beam (higher powers have a smaller diameter) directly, without any lenses in the beam path. Because the Verdi cannot be operated in a pulsed mode, a fast mechanical shutter (Newport 76992) was used to create pulses with this laser. The beam was profiled at the height of the cage floor by a Thorlabs BC106 CCD camera beam profiler. The beam profile was highly Gaussian, so the width reported was the full width at 13.5 % ($1/e^2$) of the maximum intensity. One experiment was performed with a telescope expanding the beam to a collimated beam with a 5.8 mm diameter. The maximum power delivered to the cage floor with this system was 8.5 W. A photodiode (observing a spot of light that passed through the beamsplitter) was used to monitor the laser and shutter performance in real time.

The Spectra-Physics GCR-250 is a flash lamp-pumped, Q-switched Nd:YAG laser that creates short pulses of light at 1064 nm, which can be frequency doubled to 532 nm using a removable KTP crystal. Experiments were conducted in both Q-switched (10 ns pulses) and “long pulse” (non-Q-switched, ~90 μs pulses) modes. For this laser, a digital delay/pulse generator was used to produce two pulses for Q-switched operation – one to fire the flashlamps, and one delayed by 190 ns to open the q-switch. Compared with the Verdi setup above, the optical path was longer to allow a slow focus (rather than being collimated) to a ~2.5 mm spot on the cage floor.

The 976 nm diode laser was operated with a Thorlabs ITC4020 laser diode controller. After being triggered by the pulse generator, the controller directly pulsed the diode laser, so no shutter was

needed. The optical path was similar to that for the Verdi laser, but with an 850 nm short pass filter as the dichroic beamsplitter, and a set of slow lenses to focus the multimode output to a 2.5 mm Gaussian spot at the cage floor. The measurement hardware was the same as for the Verdi V10.

All aspects of the 1470 nm diode laser setup were identical to the 976 nm diode laser, with the exception of the dichroic beamsplitter. The focusing optics were also slightly different to adjust for wavelength and different numerical apertures of the sources.

The 10.6 $\mu$m $CO_2$ laser was controlled with the Synrad UC-2000, which pulsed the laser directly while receiving a gate signal from the pulse generator. The UC-2000 was used to set the repetition rate and duty cycle of the laser, and the pulse generator set the overall pulse width. The optics used previously were replaced with gold mirrors, zinc selenide lenses and windows (latter to act as variable attenuators), and a dichroic mirror that reflects 10.6 $\mu$m while transmitting visible light (ISP Optics, BSP-DI-25-3). Spot sizes of 2.5 mm and 5.8 mm were produced and profiled with an Ophir NanoScan scanning slit beam profiler.  An infrared photodiode was used to verify pulse width.

**Supplementary Discussion S1 – Confinement zones.**

When a laser pulse is incident upon a material, photothermal and photomechanical effects may be confined to the optical zone (i.e. where photons become distributed in the irradiated material) or may be distributed outside that zone. The interplay between the optical zone and laser pulse duration is summarized by defining three regimes: stress confinement, thermal confinement, and no confinement. Stress confinement occurs when the laser pulse is so short that it deposits its energy in the optical zone before stress waves from the resulting thermoelastic expansion can dissipate out of the irradiated volume. Consequently, large stress waves are propagated, which can photomechanically ablate material within the optical zone and disrupt adjacent material outside the irradiated zone. In biological applications, stress confined pulses typically ablate the tissue they directly impact and cause mechanical damage (disrupt ordered tissues, break down extracellular matrix, etc.) in surrounding regions, whose width is on the order of the size of the incident laser spot. Thermal confinement occurs when the laser pulse is sufficiently short that all of its energy is deposited in the optical zone as heat before thermal energy can diffuse out of that zone. Heat thus accumulates efficiently during the laser pulse and high temperatures are achieved. When thermally confined pulses impact biological tissues, the irradiated area can suffer damage ranging from protein denaturation to tissue coagulation or ablation, with minimal thermal damage seen in surrounding tissue. Without confinement, thermal energy from the optical zone is able to diffuse into surrounding material during the pulse, leading to larger zones of less severe thermal damage. In biological applications, this kind of pulse is often used for coagulating a tissue or for bulk tissue removal with concomitant hemostasis, since the collateral thermal damage is sufficient to close off most blood vessels. Supplementary Fig. S1 shows a generic confinement zone plot of penetration depth (inverse of absorption coefficient) versus pulse duration, with the zone boundaries defined by properties of water.

**Supplementary Figure S1 – Confinement zone chart for water.** Typical distribution of confinement zones for water. Dashed line shows separation between pulse durations in stress confinement versus thermal confinement, as defined by penetration depth (inverse of absorption coefficient) and speed of sound in the irradiated material. Dotted line shows separation between pulse durations in thermal confinement versus no confinement, as defined by penetration depth and thermal diffusivity in the irradiated material.

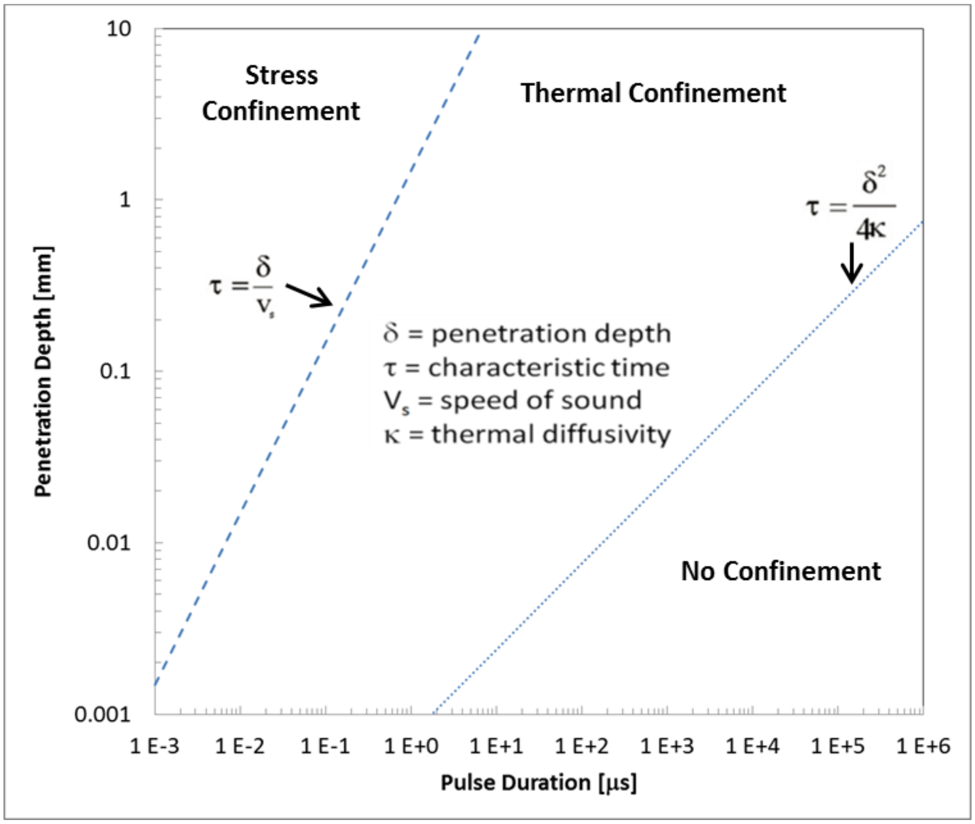

Supplement: Supplementary Information [file srep20936-s1.pdf]
